# Identification and transcript analysis of a novel wallaby (*Macropus eugenii*) basal-like breast cancer cell line

**DOI:** 10.1186/1476-4598-7-1

**Published:** 2008-01-07

**Authors:** Julie A Sharp, Sonia L Mailer, Peter C Thomson, Christophe Lefèvre, Kevin R Nicholas

**Affiliations:** 1 CRC for Innovative Dairy Products, Department of Zoology, University of Melbourne, VIC 3010, Australia; 2 CRC for Innovative Dairy Products, Biometry Unit, School of Land, Water and Crop Sciences, University of Sydney, NSW 2006, Australia; 3 Victorian Bioinformatics Consortium, Monash University, Clayton, Victoria, 3800, Australia

## Abstract

**Background:**

A wide variety of animal models have been used to study human breast cancer. Murine, feline and canine mammary tumor cell lines have been studied for several decades and have been shown to have numerous aspects in common with human breast cancer. It is clear that new comparative approaches to study cancer etiology are likely to be productive.

**Results:**

A continuous line of breast carcinoma cells (WalBC) was established from a primary breast cancer that spontaneously arose in a female tammar wallaby (*Macropus eugenii*). The primary tumor was 1.5 cm^3 ^and although large, did not appear to invade the stroma and lacked vimentin expression. The WalBC cell line was cultured from the primary tumor and passaged for 22 months. WalBC cells displayed an epithelial morphology when grown on plastic, were not EGF responsive, stained strongly for cyto-keratin and negatively for vimentin. WalBC cells were shown to be non-invasive within a Matrigel invasion assay and failed to produce tumors following transplantation into nude mice. Gene expression profiling of WalBC cells was performed using a cDNA microarray of nearly 10,000 mammary gland cDNA clones and compared to normal primary mammary cells and profiles of human breast cancer. Seventy-six genes were down-regulated and sixty-six genes were up-regulated in WalBC cells when compared to primary mammary cells. WalBC cells exhibited expression of known markers of basal invasive human breast cancers as well as increased KRT17, KRT 14 and KRT 19, DSP, s100A4, NDRG-1, ANXA1, TK1 and AQP3 gene expression and decreased gene expression of TIMP3, VIM and TAGLN. New targets for breast cancer treatment were identified such as ZONAB, PACSIN3, MRP8 and SUMO1 which have human homologues.

**Conclusion:**

This study demonstrates how novel models of breast cancer can provide new fundamental clues regarding cancer etiology which may lead to new human treatments and therapies.

## Background

Breast cancer is the leading cause of mortality and morbidity of women in many countries and is truly a multidisiplinary problem. New ways are currently being sort to develop a new focus and new perspectives. One of these methods involves the study of alternative models of breast cancer for use in comparative oncology. Comparative oncology uses breast cancer models proffered by other species in an attempt to gather more information about breast cancer which may give rise to new human therapeutic targets and interventions.

Murine, feline and canine breast cancer models have been studied extensively showing numerous commonalities with human breast cancer [1,2], [57]. Carcinomas of the mammary gland are common among carnivorous animals and although rare in herbivores, have been documented in such animals as the horse [3]. Mammary neoplasms in marsupials have not been reported to date, and interestingly, marsupials spend a relatively long period in a state of lactation.

The morphology of the wallaby mammary gland is similar to other mammals but the lactation strategy adapted by marsupials is different to eutherians. The gland undergoes lobular alveolar development during a short period of pregnancy (26.5 days) and the mother gives birth to an altricial young [4]. During a relatively long lactation the mother progressively changes it's milk composition to regulate the considerable growth and development of the young [5-8]. The mammary gland grows considerably during lactation [4] and, at the end of lactation the mammary gland undergoes involution and returns to a virgin-like state [4].

The ability to establish primary cultures of tumor cells is an important prerequisite in cancer research, allowing the study of carcinogenesis, prognostic factors and therapeutic agents [9]. In this report, a mammary carcinoma in a tammar wallaby was examined by histopathological techniques and a breast cancer cell line was established (WalBC). Transcriptional profiling using a custom tammar wallaby microarray was performed on the WalBC cell line identifying a gene expression profile consistent with human basal-like breast cancer. Moreover, in addition to the known markers for basal-like breast cancer, the WalBC cell line expressed a number of genes with homologues in the human genome, but have not previously been associated with breast cancer. Further study of these genes within human models of breast cancer may provide new clues in the development and progression of breast cancer which may in turn lead to new treatments and therapies.

## Materials and methods

### Animals, tumor detection and surgery

Tammar wallabies used in this study were part of a captive breeding colony maintained in an open enclosure at The University of Melbourne Macropod Research Facility (Melbourne, Victoria), with stock originally from Kangaroo Island, South Australia. Care and treatment of animals conformed to the National Health and Medical Research Council of Australia and were approved by the Victorian Department of Sustainability and Environment Ethics Committee. The animal presenting with a mammary lesion was part of the breeding colony for two years (February 2002 – February 2004) and was checked monthly during this time for signs of mating and presence of pouch young. The animal did not reproduce in this time but presented with a mammary lesion. The animal was subsequently monitored weekly for three weeks then euthanized. The lesion was measured, excised and either immediately frozen for future RNA extraction, placed in Hanks' Balanced Salt Solution (HBSS) (Sigma Aldridge, Sydney, Australia) at 4°C in preparation for isolation of mammary cells or fixed in formalin for histology. The tissue from the mammary gland was divided into two portions, one comprising the main body of the mammary lesion and the other comprising normal mammary tissue. Mammary gland tissue was also collected from a wallaby of comparable age and reproductive status for preparation of mammary epithelial cells.

### Preparation of WalBC cell line and wallaby primary mammary epithelial cells

Tissue was immediately transferred to 1× Hanks' Balanced Salt Solution (HBSS) (Gibco, USA) with 20 μL/mL penicillin/streptomycin (Gibco, USA) and 5 μg/mL Fungizone (Gibco, USA) on ice and transported back to the laboratory for enzymatic digestion to harvest mammary epithelial cells.

Tissue was dissected free from fat, weighed, sliced finely and digested with 1 μg/mL Collagenase Class2 (Worthington UK), 10 mL/L penicillin/streptomycin (Gibco, USA), 10 mL/L fungizone (Gibco, USA), 0.35% Bovine Serum Albumin (Sigma) and a final concentration of 0.5 mM glucose. 10 g of tissue per 100 mL of media was digested shaking at 200 rpm at 37°C for 4 h. Cells were harvested by filtration through 150 μm nylon mesh using a Nalgene filter unit. The suspension was centrifuged at 80 g for 5 min and pellets were washed twice with HBSS containing 0.02 mg/mL DNase 1 (Invitrogen) and 1 mg/mL Trypsin Inhibitor (Sigma). Cells were again suspended in wash media, then filtered through 53 μM nylon mesh, re-centrifuged and finally resuspended in FCS/10%DMSO (DMSO-Sigma, Sydney, Australia), and frozen at a density of ~2 × 10^7 ^cells/mL.

WalBC cells and wallaby primary mammary cells were cultured in either 25 or 80 cm^2 ^culture flasks in 10 mL or 20 mL respectively of M199/Hams/Hepes media with 1 μg/mL cortisol, 10 ng/mL EGF, 1 μg/mL insulin supplemented with 10% fetal bovine serum. WalBC cells were passaged (more than 20 times) when confluent by scraping and use of versine solution in phosphate buffered saline (PBS) (Sigma-Aldrich, Sydney, Australia). Passaging of cells with 0.1% trypsin-versine in phosphate buffered saline (PBS) (Sigma-Aldrich, Sydney, Australia) for 2 minutes at 37°C disrupted cellular aggregates but cells failed to retain viability following this treatment.

### Histology

Tissue was fixed in 10% formalin for 24 h. Samples were processed (Citadel; Shandon Scientific Ltd., Cheshire, England), and embedded in paraffin using routine procedures. Paraffin-embedded sections of 5 μm thickness were cut, mounted on 3-aminopropyltriethoxysilane-coated slides and submerged in histolene to remove the paraffin. After rehydration, sections were stained with haematoxylin and eosin. Finally, sections were coverslipped and examined using an Olympus BX40 microscope and Coolscope digital microscope (Nikon) for light microscopy.

### Immunohistochemistry

Paraffin-embedded sections of 5 μm thickness were cut and mounted on 3-aminopropyltriethoxysilane-coated slides and submerged in histolene to remove the paraffin. After rehydration, tissue peroxidases were blocked for 30 min with a 1% hydrogen peroxide solution and washed with 1× PBS. For detection of Vimentin, sections underwent treatment with sodium citrate buffer. Sections were then blocked for 60 minutes with 10% goat serum (Sigma)/1% BSA/PBS prior to addition of Vimentin antibody (V9, 1:800; Dako, Australia) at 4°C overnight. An HRP-conjugated goat anti-mouse secondary antibody (1:250 dilution; Dako, Australia) was then applied for 1 hour to allow a brown precipitate to develop using AEC (Dako, Australia). Finally, sections were counterstained with eosin, dehydrated, coverslipped and examined using a Coolscope digital microscope (Nikon) for light microscopy.

### Proliferation assay and cell morphology

WalBC or wallaby MEC's cells were plated (2000 cells/well) in 96 well plate formats with growth media containing either insulin (I; 1 μg/ml), cortisol (F; 1 μg/ml) and prolactin (P; 1 μg/ml) or I, F, Epidermal growth factor (EGF; 10 ng/ml). Cells were grown for a further 10 days before being fixed and stained with Sulforhadamine B as previously described [10]. Each time point was performed in quadruplicate. Cells were visualized by phase contrast microscopy using an Olympus BX40 microscope and photographed using a DigitalSight DSL1 (Nikon) camera.

### Matrigel outgrowth assay

Matrigel outgrowth assays were performed in 48 well plates as previously described (Price and Thompson, 1999). Cells (2 × 10^4^) were dispersed in 75 μL of undiluted Matrigel (approx.10 mg/mL) and then overlaid onto 100 μL of polymerized undiluted Matrigel. Once the top layer had polymerized the cultures were incubated in MEM/10%FBS media for up to 10 days and photographed at 20 × magnification by phase contrast microscopy using an Olympus BX40 microscope and photographed using a DigitalSight DSL1 (Nikon) camera.

### In vivo studies

Mice (3–4 week old intact female Balb/C *nu/nu*) were purchased from Australian Resource Center (Perth, Australia), housed in individually ventilated cages under filtered air (Techniplast, Milan, Italy) and acclimatized for one week prior to manipulation. Anesthesia was achieved by i.p injection of ketamine/xylazine (Provet; Australia; 40 μg/g mouse and 16 μg/g mouse, respectively). The mice were allowed to recover from the anesthesia before being returned to their cages and monitored daily. Animal studies were conducted with ethical approval of the St. Vincent's Hospital Animal Ethics Committee (Melbourne, Australia), and in accordance with the Australian National Health and Medical Research Council's Guidelines for the Care and Use of Laboratory Animals.

### Mammary fat pad inoculation of WalBC cells

WalBC cells were harvested from near confluent conditions, aspirated into cell suspension (cell aggregates and single cells were present), washed thrice and resuspended in PBS before inoculation. Two groups of 8 mice received mammary fat pad inoculation of WalBC cells (5 × 10^5 ^cells/15 μL) as described by Price et al [11]. Tumor growth was assessed by monitoring the fat pad for palpable tumors at weekly intervals. Mice were sacrificed after 6 months.

### Immunocytochemistry

WalBC cells grown on plastic were fixed in 4% paraformaldehyde (30 min) and washed three times in PBS. Cells were permeabilized with 0.1% Triton X/PBS (5–10 min) and washed thrice with PBS before blocking in 1% BSA for 30 min. Either Vimentin9 (Dako; 1/800) or Rabbit anti-cytokeratin (Bectin Dickinson1/400) antibody was added in blocking buffer and incubated overnight at 4°C. Cells were then washed five times in PBS to remove non-specific binding of primary antibody. Cells were then incubated in goat anti-mouse (FITC) (DAKO; 1/400) or goat anti-rabbit (FITC) (DAKO; 1/400) in blocking buffer for 1 hour and washed thrice with PBS. Nuclei were visualized using Propidium Iodide (Invitrogen). Images were visualized for fluorescence using an Olympus BX40 microscope and photographed using a DigitalSight DSL1 (Nikon) camera.

### RNA preparation for gene expression by microarray analysis

WalBC cells, primary mammary cells cultured from the same animal and primary wallaby mammary cells cultured from a virgin animal of similar age were scraped from tissue culture dishes using Tripure (Roche). Total RNA was isolated from the aqueous phase and further purified using the Qiagen RNeasy miniprep kit (Sydney Australia) following the manufacturer's instructions.

RNA Amplification was done in 3 parts similarly to the Eberwine protocol [12]. First strand synthesis utilized MMLV RNase H- (Promega M3681) and second strand synthesis was done with DNA Polymerase 1 (Promega, M2501). Lastly *in vitro *transcription was performed with the T7 Megascript Kit (Ambion 1334). The resulting amplified RNA was then further purified using the QIAGEN RNeasy miniprep kit.

The amplified RNA from each treatment group was labeled using amino allyl reverse transcription followed by Cy3 and Cy5 coupling. Samples of amplified RNA (10 mg) were reverse transcribed using 5 μg random hexamers (Geneworks), MMLV reverse transcriptase (Promega), RNAse H (Invitrogen) and 1× buffer at 42°C for 2.5 hours. The reaction mix was hydrolyzed by incubation at 65°C for 15 minutes in the presence of 55 mM NaOH, 55 mM EDTA followed by a subsequent addition of acetic acid to 50 mM. The cDNA was then adsorbed to a Qiagen QIAquick PCR Purification column. Coupling of either Cy3 or Cy5 dye was performed on the column by incubating the adsorbed cDNA with the appropriate dye in 0.1 M sodium bicarbonate pH 9.0 for 1 hour at room temperature in darkness. Each labeled cDNA was eluted in 80 μl of water and was then combined with its comparing sample during further purification on a second Qiagen QIAquick PCR Purification column. The joint Cy3 and Cy5 labeled probe in a final concentration of 0.4 mg/ml yeast tRNA, 1 mg/ml human Cot 1 DNA, 0.2 mg/mL Poly dA_50_, 1.25 × Denharts, 3.2 × SSC and 50% formamide was heated to 100°C for 3 minutes. SDS, to 0.1%, was added immediately after heating and just prior to application. Probes were hybridized to custom made tammar wallaby EST microarray slides overnight at 42°C in a HyPro20 (Integrated Science) humidified chamber. The slides were printed with 10,000 EST's from tammar mammary gland cDNA libraries generated from tissue collected across the lactation cycle (Lefevre, manuscript in preparation).

The tammar EST database was derived from several cDNA libraries comprising day 23 pregnant (n = 4), lactating at day 130 (n = 4), lactating at day 260 (n = 1), lactating at day 130 subtracted for all the major milk protein genes (n = 2), non-lactating (n = 2) and a normalized library (combined RNA from day 26 pregnant, lactating at day 55, day 87, day 130, day 180, day 220, day 260 and involuting at day 5).

Microarray's were washed in 0.5× SSC, 0.01% SDS for 1 minute, 0.5× SSC for 3 minutes then 0.006× SSC for 3 minutes at room temperature in the dark. Slides were centrifuged dry at 130 g for 5 minutes then scanned with a VersArray Scanner (BioRad). Images were analyzed using Versarray Software (Biorad).

### Analysis of gene expression data

Gene expression data was normalized using the single channel normalization method in the Limma package of Bioconductor [13]. These normalized expression values were analyzed using a two-stage process [14]where all the expression values are considered simultaneously. The first stage involves fitting a linear mixed model [15]of the form

M_adj _= μ + Treatment + Probe + Treatment.Probe + ε

where M_adj _is the adjusted (loess-normalized) log intensity ratio for a probe on the cDNA array, Treatment is the fixed effect of the treatment (tumor cells, non-tumor cells, or virgin), Probe is the random effect of the probe on expression levels, regardless of the treatment, and Treatment.Probe is the random effect of a particular probe within a particular treatment, the effect of interest. Typically, the distribution of these random Treatment.Probe effects show a mixture of two distributions, one with small variance (non-differentially expressed genes, non-DE) and one with large variance (differentially expressed, DE). So the second stage involved fitting a two-component mixture model (DE vs non-DE) to these effects [16], and this will return the (posterior) probability that a particular gene is DE, given its Treatment.Probe effect, with a probability in excess of 0.5 indicating a gene is more likely DE, for that particular treatment. However, a stringent threshold has been used requiring a posterior probability in excess of 0.999 before a gene was classified as being DE, in order to reduce the false positive rate. The statistical package R was use for this two-stage process.

### Statistical analysis

To determine the significance of EGF response within each cell type a paired students t-test was performed on quadruplicate samples at day 10. For Matrigel outgrowth, 20 outgrowths were measured for each cell type at day 4 in a given area and averaged. Unpaired t-test was used to determine significance differences in the length of outgrowths between the two cell types.

## Results

### Pathology and immunoreactivity of a wallaby primary tumor

**Figure 1:**
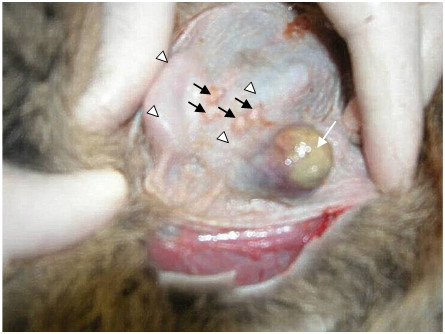
*In situ *wallaby breast tumor. A 1.5 cm^3 ^breast lesion (white arrow) was identified within the left posterior mammary gland of a non-lactating mature female tammar wallaby. Mammary glands are indicated by white arrowheads and teats are indicated by black arrows. Skin has been cut away and pulled back to expose the area.

**Figure 2:**
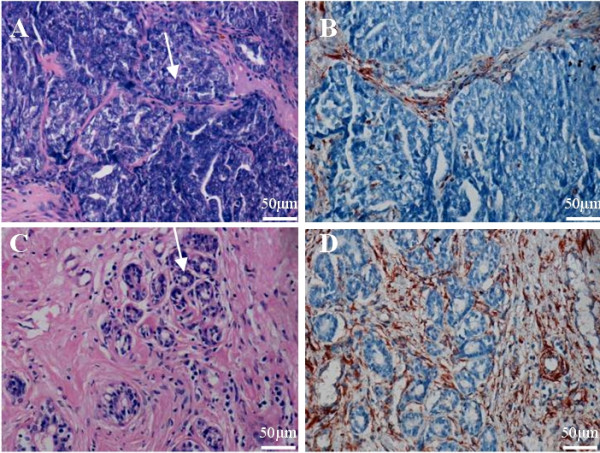
Histological analyses of normal tammar wallaby mammary gland and primary breast cancer from the same animal. (A) H & E staining of wallaby carcinoma within breast tissue showing solid tumor architecture with an invading margin. Basophilic tumor cell nests are stained blue and are indicated by the arrow. (B) Vimentin staining shows cancer cells are vimentin (V9) negative, while vimentin expression can be identified (brown) within the stromal tissue. (C) H &E staining of normal tammar wallaby mammary gland showing alveolar structures (arrowed) and (D) stroma staining of vimentin (V9) (brown). Scale bars are shown.

A 1.5 cm^3 ^mammary lesion was detected in a female, non-pregnant, non-lactating tammar wallaby (Fig. 1). The female was not less than two years of age, although her true age was not determined as she was captured from the wild and held in a captive colony for two years prior to tumor presentation. Examination by histological techniques showed primary tumor cells were present within the gland arranged in solid masses which appeared to display a pushing margin that invaded the stroma (Fig. 2A). Anti-vimentin immunostaining revealed positive vimentin immunoreactivity of the stroma and negative immunoreactivity of the tumor cells (Fig. 2B).

### Characterization of the WalBC cell line: Morphology, immunoreactivity and growth/invasive potential

**Figure 3:**
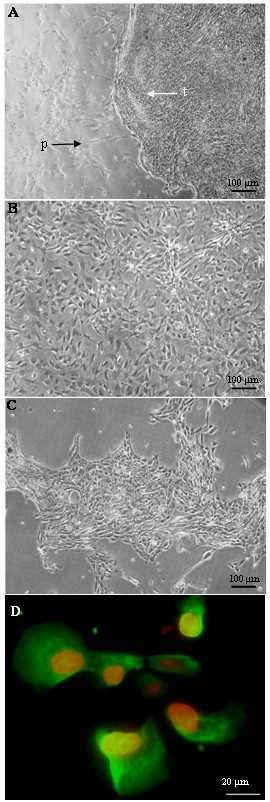
Cell morphology in *in vitro *culture. (A) Initial culture of mammary tumor showed two cell types growing within the flask. Continued culture allowed primary mammary cells (indicted by p) to die off leaving only the tumor cells (indicated by t). (B) Primary mammary wallaby cells and (C) established wallaby breast cancer cell line (WalBC) after passaging for 22 months grown on tissue culture treated plastic. (D) WalBC cells exhibited positive cytoplasmic staining for cytokeratin (green). Cell nuclei are stained red using propidium iodide. Scale bars are shown.

The WalBC cell line was grown from a mixture of cells containing both normal mammary stromal/epithelial cells and primary cancer cells (Fig. 3A). The WalBC cell line formed a monolayer comprising a homogeneous cell population which exhibited epithelial morphology and strong cell-cell interactions. Once established, the WalBC cell line was passaged for 2 years, cells maintained a cuboidal morphology and proliferated to islands of confluent cells. Single cells did not survive in the absence of cell contact and passaging of cells required the presence of large cellular aggregates which attached to the tissue culture treated plastic before initiating proliferation. Use of trypsin for passaging was unsuccessful due to the break down of cellular aggregates. The morphology of wallaby primary cells and WalBC cells grown in culture was markedly different with WalBC cells appearing smaller and exhibiting strong cell-cell contact (Fig. 3B, C).

Immunostaining with anti-cytokeratin of WalBC cells grown in culture showed 90% of cells exhibited strong cytokeratin immunoreactivity which localized to the cytoplamic region of positive cells (Fig. 3D). A small population of cells showed weaker cytokeratin immunoreactivity, however all cells appeared to show some degree of immunoreactivity.

**Figure 4:**
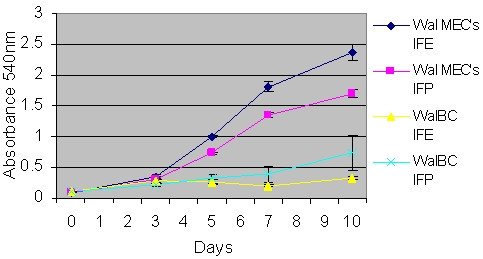
Comparative analysis of proliferation rates between WalBC and wallaby MEC's. Cells were grown for 10 days in the presence of insulin (I), cortisol (F) and prolactin (P) or I, P and epidermal growth factor, EGF (E). Wal MEC's responded to the presence of EGF (P = 0.0041, 95% confidence interval, t = 8.0113, df = 3).

The proliferation rate of the WalBC cell line was compared to primary wallaby epithelial cells (MEC's) in the presence of insulin (I), cortisol (F) and prolactin (P) or I, F and epidermal growth factor (EGF). Wallaby MEC's exhibited a higher proliferation rate compared to WalBC (Fig. 4). Wallaby MEC's also showed an increase in proliferation in the presence of EGF compared to growth in the presence of prolactin (P < 0.01) while the WalBC cell line failed to respond to EGF treatment.

**Figure 5:**
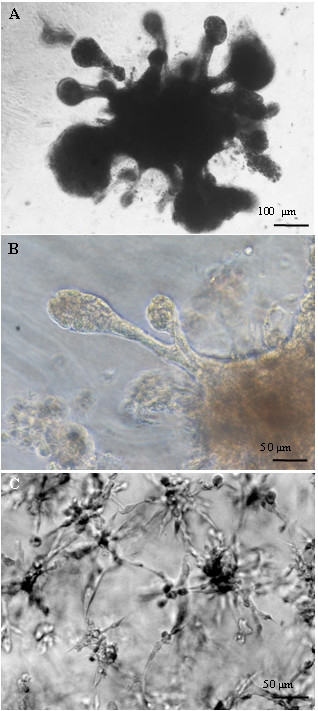
Comparison of invasive potential of wallaby and human breast cancer cell lines. The wallaby breast cancer cell line, WalBC and human breast cancer cell line, MDA-MB-231 were grown within Matrigel (4 days). (A, B) WalBC cells exhibit development of mammary-like structures suggestive of a non-invasive phenotype and (C) MDA-MB-231 cells exhibit stellate outgrowth suggestive of an invasive phenotype.

The invasive potential of the WalBC cell line was examined by Matrigel invasion assay. This assay has previously associated stellate Matrigel morphology with invasiveness in human breast cancer cell lines [11,17]. The WalBC cell line failed to exhibit stellate growth but grew mammary-like structures that resemble ducts and lobules (Fig. 5). These structures also mimicked the morphology exhibited by the *in situ *primary lesion which showed masses of tumor cells surrounded by stromal cells. Matrigel morphology analysis was also performed on a known human invasive breast cell line, MDA-MB-231 which demonstrated stellate outgrowth as expected. Comparative quantitative analysis was performed to determine the average length of outgrowth between WalBC and MDA-MB-312 cells. The average outgrowth for WalBC cells in Matrigel was found to be 145 μm (standard deviation of the mean = 72.147) and MDA-MB-231 cells showed an average outgrowth of 50.75 μm (standard deviation of the mean = 20.601). The difference between the length of outgrowths between the two cell types was determined to be highly significant (P < 0.0001). An *in vivo *model of tumor growth in nude mice was used to study the degree of tumorgenicity of the WalBC cell line. WalBC cells were tested on two separate occasions using two groups of eight mice and a different WalBC passage numbers and on both occasions WalBC cells failed to generate palpable tumors (data not shown).

### WalBC gene expression profile

The gene expression profile of the WalBC cells was compared to primary mammary epithelial cells (MEC's) from the same animal and virgin mammary epithelial cells grown from a different animal using a microarray with 10,000 tammar mammary ESTs [18]. Genes were considered differentially expressed if there was a 2-fold or more increase or decrease of intensity between WalBC and both normal MEC's from the same animal and virgin MEC's from a different animal, with a posterior probability of >0.999. Gene expression profiles revealed a large number of differentially expressed genes were associated with carcinogenesis (Table 1 and 2). The highest up-regulated gene in the WalBC cell line was cytokeratin 17 while vimentin was down regulated. Notably WalBC cells showed up regulated expression of DSP, s100A4, ANXA1, NDRG-1, TK1 and down regulation of genes such as tissue inhibitor of TIMP-3 and TAGLN. The overall expression profile revealed a pattern of gene expression consistent with basal-type breast cancer. A number of EST's were also identified that have not been previously associated with breast cancer. These included CAP43, SALL1, ZONAB, SUMO-1 and MRP8 and a number of hypothetical proteins with homologues within the human genome.

**Table 1:** Up-regulated genes in WalBC cell line.

Gene	+ve Fold change^1 ^c/w MEC's	+ve Fold change^1 ^c/w virgin MEC's	Wallaby array	Human Unigene
^2 ^KRT17	30.073	5.751	SGT20f3_B03	Hs.2785
^2 ^KRT14	26.983	3.388	SGT20m2_B01	Hs.355214
unknown	16.757	2.863	SGT20s4_B07	
^2 ^DSP	15.967	4.621	SGT20w1_H09	Hs.349499
^2 ^S100A14	13.558	4.540	SGT20j6_D06	Hs.288998
^2 ^*FABP5*	11.835	4.202	SGT20k1_B09	Hs.414321
KERV-1	11.131	6.870	SGT20r1_G05	
PIGPC1	8.865	4.520	SGT20u3_F08	Hs.303125
^2^KRT17	8.671	4.912	SGT20t2_C06	Hs.2785
DZ-HRGP	8.033	2.635	SGT20h1_E03	Hs.330537
SNK	7.208	4.010	SGT20r1_E01	Hs.375912
^2^AQP3	5.941	8.356	SGT20l2_D09	
^2 ^NDRG1	5.596	3.378	SGT20c1_B05	Hs.75789
^2^TK2	5.430	3.310	SGT20u4_B02	Hs.105097
SALL1	5.315	2.024	SGT20h1_C08	
^2 ^EIF5A	5.296	3.989	SGT20i3_B10	Hs.381006
TP73L	4.920	3.483	SGT20v4_H01	
^2^ANXA1	4.822	2.234	SGT20n1_D06	Hs.78225
MET	4.790	2.524	SGT20h2_E01	
^2 ^MeCP2	4.634	3.279	SGT20f1_G03	Hs.25674
HGNC	4.483	2.218	SGT20o1_G06	Hs.331195
^2 ^PTPN12	4.477	2.903	SGT20i4_F05	Hs.62
^2 ^MeCP2	4.261	3.164	SGT20v3_G09	Hs.25674
^2^CST6	4.189	2.143	SGT20n1_F03	Hs.83393
UCH-L3	4.184	2.167	SGT20m2_G11	Hs.77917
HERV-K pol	4.144	3.806	SGT20j6_F04	
Hs3st3b	4.067	2.765	SGT20l2_F04	Hs.8040
AP1M2	4.021	2.062	SGT20h4_C03	Hs.18894
W86 retroposon	3.890	2.403	SGT20k3_A02	
Envelope protein	3.859	2.167	SGT20o3_C12	
STK24-like	3.764	2.017	SGT20j6_C11	Hs.168913
MRP8	3.530	2.085	SGT20w4_B01	Hs.335891
BDP1	3.476	2.914	SGT20q4_C12	Hs.380461
RP1-119E23 on chromosome Xq25-27.1	3.386	2.290	SGT20l1_B06	
^2 ^ANXA1	3.254	3.022	SGT20k2_A08	Hs.78225
MYH7	3.210	2.197	SGT20u4_B10	
RIKEN cDNA 0610009O03 gene	3.138	2.511	SGT20k3_E06	Hs.157145
Hypothetical protein XP_148915	3.110	2.333	SGT20h4_C11	Hs.385695
^2 ^Cap43	3.102	2.243	SGT20r4_B01	Hs.381559
^2^HSPA1A	2.998	2.406	SGT20d4_F03	Hs.80288
^2^CK19	2.977	2.120	SGT20o4_G10	Hs.182265
MYO10	2.737	2.110	SGT20f3_B11	Hs.61638
Sumo-1	2.728	2.646	SGT20v3_B08	Hs.81424
^2 ^NPKC-ZETA	2.701	3.062	SGT20k4_H11	Hs.78793
ZONAB	2.639	2.321	SGT20c5_F02	Hs.198726
14-3-3 zeta	2.632	2.117	SGT20n4_D05	Hs.75103
BAC clone RP11-391A7	2.721	2.307	SGT20k3_E11	
Hypothetical protein DKFZp434H2035.1	2.589	2.477	SGT20i6_H04	Hs.381781
ATDC	2.547	2.148	SGT20q3_F11	Hs.82237
RP11-554F11 on chromosome 10	2.528	2.010	SGT20d5_G01	
^2 ^HSPB1	2.516	2.761	SGT20k3_G03	Hs.385457
SPC18	2.466	3.827	SGT20u4_A12	Hs.68644
Replicase	2.465	2.009	SGT20i5_D11	
gp1	2.455	2.378	SGT20i5_B12	
^2^KRT14	2.379	2.583	SGT20d5_B06	Hs.355214
ITMCII-3b	2.370	2.042	SGT20e2_F01	Hs.433982
Hypothetical protein	2.361	2.762	SGT20l2_F09	
LAT	2.354	2.085	SGT20q3_F05	
protein for MGC:33586	2.279	2.855	SGT20i3_H10	Hs.348516
SBDS	2.226	2.778	SGT20e2_G04	Hs.110445
^2^AHA1	2.179	2.169	SGT20s4_B04	Hs.204041
3 BAC RP11-641D5	2.172	2.652	SGT20i6_A03	
DKFZp667G2110	2.164	2.725	SGT20q4_B10	Hs.406105
UBE2C	2.146	2.374	SGT20r5_F02	Hs.26213
SLC3A	2.091	2.120	SGT20o5_D01	Hs.79748
^2^CLDN4	2.030	3.757	SGT20f4_E07	Hs.5372
PACSIN3	2.008	2.504	SGT20t1_F02	Hs.334639

^1^P > 0.999 and > 2 fold

^2 ^Previously associated with breast cancer

**Table 2:** Down-regulated genes in WalBC cell line.

Gene	-ve Fold change^1 ^c/w MEC's	-ve Fold change^1 ^c/w virgin MEC's	Wallaby array	Human Unigene
^2^TAGLN	28.315	12.585	SGT20p3_D11	Hs.433399
GSTP1	24.654	10.312	SGT20b1_E11	Hs.226795
CYR61	23.276	20.831	SGT20l1_B02	Hs.8867
PCPE	21.766	12.276	SGT20v3_A09	Hs.202097
CLU	14.308	19.773	SGT20k1_D12	Hs.433909
OPG	14.182	2.245	SGT20h4_G04	Hs.81791
LTBP-1	12.281	3.654	SGT20u5_B04	Hs.241257
COL11A2	12.076	13.887	SGT20s4_B10	Hs.179573
ZP3	11.225	14.406	SGT20c5_D09	
STEAP1	10.141	3.472	SGT20h4_D01	Hs.61635
SORBS2	9.922	5.057	SGT20p1_G06	Hs.379795
FCRN	9.755	4.917	SGT20h3_H02	
IGFBP6	9.163	6.379	SGT20n2_E09	Hs.274313
Facl4	8.656	3.001	SGT20j3_E06	Hs.81452
NGAL	8.593	10.225	SGT20n2_B01	
ANXA6	8.269	4.562	SGT20p1_E11	Hs.118796
AEBP1	7.498	4.536	SGT20t5_H04	Hs.118397
GSTP1	7.319	4.592	SGT20t5_E05	Hs.226795
EFEMP2	7.254	2.088	SGT20i6_G04	Hs.381870
HGNC	6.631	2.603	SGT20i4_F11	Hs.433706
OXCT1	6.284	2.334	SGT20s4_D01	Hs.177584
SERPINF1	6.192	2.368	SGT20p2_D03	Hs.173594
ACTA2	6.170	4.074	SGT20l2_G07	Hs.195851
AEBP1	5.010	3.155	SGT20w2_B10	Hs.118397
EFEMP2	4.967	2.226	SGT20c5_E02	Hs.6059
MAN2B1	4.852	3.721	SGT20k3_F10	Hs.381666
FLJ23614	4.749	2.496	SGT20p3_C10	Hs.28780
^2^VIM	4.400	4.844	SGT20k1_E07	Hs.297753
FLJ20421	4.363	2.126	SGT20i3_D01	Hs.378857
FBLN1	4.113	2.024	SGT20s4_E03	
FABP3	4.078	2.829	SGT20s2_D02	Hs.49881
FLNC	4.065	2.108	SGT20k1_D09	Hs.58414
RP23-38K18 on chromosome 4	3.983	2.206	SGT20h4_B11	
LRP1	3.894	2.376	SGT20s4_D06	Hs.89137
CJS1	3.938	2.187	SGT20h1_E08	
EPB41L1	3.502	2.668	SGT20n2_B11	Hs.253756
MGC10731	3.497	2.873	SGT20c5_B09	Hs.322487
G6PD	3.458	2.366	SGT20r1_D08	Hs.80206
EMP3	3.439	2.614	SGT20t2_E08	Hs.76884
ID2	3.424	3.370	SGT20v5_C11	Hs.9999
FTH1	3.340	2.057	SGT20j1_E06	Hs.431709
^2^CTSZ	3.316	2.207	SGT20h4_E12	Hs.252549
COL6A1	3.261	5.085	SGT20u4_F02	Hs.108885
HGNC	3.238	3.114	SGT20i2_H07	Hs.283611
CTSD.	3.149	3.815	SGT20q1_A04	Hs.343475
VIP36	3.127	4.497	SGT20k3_D10	Hs.75864
P0514G12.26	3.110	2.124	SGT20n3_A08	Hs.368364
IMAGE3455200	3.031	2.166	SGT20r5_G10	Hs.425727
NPC2	2.981	2.165	SGT20e2_C10	
SND1	2.934	2.001	SGT20e3_A09	Hs.79093
C4bp	2.903	2.104	SGT20i5_F10	
^2^TIMP3	2.807	2.850	SGT20o1_A12	
ZFP135	2.773	2.451	SGT20b1_F01	Hs.20848
TNC	2.772	6.720	SGT20d3_B07	Hs.289114
CRP1	2.646	2.380	SGT20l4_A03	Hs.108080
EF-1-gamma	2.615	2.379	SGT20u5_G11	Hs.256184
IQGAP3	2.611	2.136	SGT20k4_D12	Hs.78993
CPT II	2.576	2.033	SGT20s5_G03	Hs.274336
6PGD	2.495	2.522	SGT20p4_H07	Hs.392837
IGFBP2	2.421	2.249	SGT20f3_D09	Hs.433326
KIAA0627	2.417	2.164	SGT20w3_G03	Hs.108614
FKBP11	2.410	2.082	SGT20s4_F11	Hs.24048
FBLN1-D	2.392	2.902	SGT20t2_F03	Hs.79732
ZFP135	2.302	2.175	SGT20l1_H06	Hs.146854
BC012173	2.268	2.078	SGT20n4_B12	Hs.7307
ALDH7A1P1	2.214	3.149	SGT20n3_D11	Hs.76392
ALG6	2.177	2.145	SGT20d4_H03	
FTL	2.156	3.180	SGT20q3_E06	Hs.433670
BC024814	2.115	2.158	SGT20h4_H07	Hs.165428
Col3A1	2.100	2.610	SGT20c4_F08	Hs.119571
SCP2	2.098	3.386	SGT20p2_H06	Hs.75760
GADD45B	2.071	4.582	SGT20k1_G01	Hs.110571
LRP1	2.042	2.326	SGT20n2_A03	Hs.251337
CD63	2.035	3.965	SGT20m3_G06	Hs.433996
dolichyl-P-Glc	2.015	2.653	SGT20m5_C11	Hs.77575
ACAT1	2.003	2.564	SGT20p4_B02	

^1^P > 0.999 and > 2 fold

^2 ^Previously associated with breast cancer

## Discussion

A wide variety of animal models have been used to study human breast cancer [19]. For example, murine, feline and canine mammary tumor cell lines have been studied for several decades and have shown to have numerous aspects in common with human breast cancer [1,2,20]. We present here the first reported discovery of a primary breast lesion in a marsupial and the subsequent establishment and characterization of the first wallaby breast cancer cell line for comparative analysis of breast cancer. The primary lesion lacked vimentin expression and the cell line was shown to be cytokeratin positive which is consistent with basal type invasive carcinoma.

In all species studied extensively so far, the ability of invasive tumor cells to interact specifically with, and invade, the extracellular matrix (ECM) has been linked to breast cancer progression [21-23]. From these studies it can be concluded that malignant progression is a stepwise process and tumor growth occurs after a series of molecular events that parallel morphological changes indicative of cell transformation. It has been well established that the invasive capability of a breast cancer cell line can be accurately predicted by performance in an *in vitro *three dimensional Matrigel outgrowth assay [17,24-29]. When placed on reconstituted basement membrane (Matrigel) breast cancer cell lines with the potential to metastasize have been shown to demonstrate stellate branching morphology and invade the matrix, while non-invasive cell lines fail to grow or develop into spherical bunches of cells without invading the matrix [17]. This *in vitro *phenomenon is thought to mimic the *in vivo *interaction of tumor cells with the surrounding matrix and demonstrates the ability of the tumor cell to degrade the basement membrane, which encapsulates the primary tumor, and allows individual cells to migrate from the initial tumor mass into the breast stroma and eventually establish within the lymph gland which aids dispersal to other organs of the body. In stark contrast, normal mammary epithelial cells form polar acini, similar to alveoli breast structures when plated on a layer of Matrigel [30-32]. The WalBC cells appeared to exhibit a non-invasive phenotype, however the growth pattern in Matrigel did not resemble the spherical bunches of cells exhibited by other non-invasive cell lines [17] but appeared to exhibit a normal branching and lobular development. This difference in morphology could be due to difference in cellular signaling via the components within Matrigel. It has been shown that mammary epithelial cells exhibit species-specific cell interaction with their surrounding extracellular matrix. Fur seal (*Arctocephalus pusillus pusillus*) mammary epithelial cells only form acinar structures capable of expressing milk protein genes when grown on their own matrix and display invasive morphology when grown on Matrigel [33]. A similar effect is also seen with primary wallaby mammary cells, which exhibit stellate outgrowth on Matrigel and only form acinar structures capable of producing milk when grown on their own matrix (Mailer and Nicholas unpublished data). These observations suggest Matrigel may not be an adequate substrate to test the invasive potential of wallaby breast cancer cell lines and a wallaby derived ECM would be better suited to use with these cells as it has the potential to more closely mimic the *in vivo *environment. Similarly, the failure of WalBC cell to establish tumorgenicity using the nude mice xenograph model may also be subject to the same species specific restraints.

Epidermal growth factor (EGF), a polypeptide found in human and animal blood and secretions, is an important mitogen in breast epithelial cells. EGF has been found to stimulate a variety of tissues including normal rodent breast tissue and rodent breast cancer [34] and human breast epithelial cells in culture [35] and fibridomas [36]. In MCF-7 cells as little as 0.01 ng/ml of EGF stimulates cell growth and 10 ng/ml was maximal, however, EGF shows no effect on another human breast cancer cell line, MDA-MB-231. [37]. Similarly, primary wallaby epithelial cells shows a growth response to EGF while the WalBC cell line was not EGF responsive as these maximal doses. It is suggested that some breast cancers retain EGF sensitivity observed with nonmalignant mammary cells, while it is lost in others. The slow growth rate observed for the WalBC cell line compared with the primary cells may indicate that this cell line requires other hormones/factors for maximal growth.

Newly emerging data about the genomes of other species such as the marsupial and the availability of 15,000 breast specific wallaby cDNA's expressed at all stages during the lactation cycle, and a 10, 000 ESTs array offers the rare opportunity to profile the WalBC cell line for gene expression patterns. Variations in transcriptional programs account for much of the biological diversity of human cells and tumors. Despite this molecular diversity, analyses of invasive breast carcinomas using microarrays have identified gene expression signatures that characterize many of the essential qualities important for biological and clinical classification [38]. DNA microarray profiling studies on breast tumors show distinct and reproducible subtypes of breast carcinoma associated with different outcomes. Expression profiles have characterized invasive breast carcinomas into five groups: luminal A, luminal B, HER2+/estrogen receptor (ER)-, basal-like, and normal breast-like. The basal-like is typically ER- and HER2- and shows some characteristics of breast myoepithelial cells. The basal-like subtype has been shown to have the highest proliferation rates and poorest outcomes [39,40], and has been described in association with BRCA-1-associated carcinomas [41]. Myoepithelial cells typically express cytokeratin 17, while luminal cells typically express cytokeratins 8 and 18. The prevalence and poor prognosis of basal-like breast carcinomas have been validated immunohistochemically; in a 564-case tissue microarray, it was demonstrated that 16% of tumors stained positive for cytokeratin5/6 or cytokeratin 17 and that basal cytokeratin expression was associated with a poor prognosis [42].

The WalBC transcript profile was compared to normal wallaby mammary cells obtained from the same animal and to mammary cells obtained from a virgin animal. Microarray analysis revealed that KRT17 (30 fold up regulated) was the highest up-regulated gene in the WalBC cell line when grown as a mono-layer culture. Expression of this gene is characteristic of basal carcinomas [42]. A 4.7 fold down regulation of VIM expression and absence of vimentin immunoreactivity in the *in situ *tumor compared to normal mammary cells suggests both the tumor and the derived cell line have not undergone an epithelial-to-mesenchymal transition associated with increased invasive/migratory properties of epithelial cells [43,44]. WalBC cells showed regulated expression of DSP, s100A14 and ANXA1 which are genes associated with well-differentiated, epithelioid breast cancer cell lines with weak invasive potential and poorly invasive tumors [45-47]. WalBC cells also over expressed the tumor metastasis suppressor NDRG1, which has been shown to be negatively correlated with tumor metastasis. *In vitro *and *in vivo *studies have also demonstrated a significant reduction in the metastatic ability of cells over-expressing NDRG1[48]. The morphology of the *in situ *tumor also resembled a basal-like carcinoma [49] which exhibited solid architecture and a pushing margin.

In addition to exhibiting a gene expression profile that correlates with poorly invasive breast cancers the WalBC cell line also displayed a gene profile that correlated with highly invasive cells. For example the up-regulation of TK1 seen in WalBC cells has previously been associated with high proliferation activity and invasiveness potential which is related to a more aggressive phenotype [47]. Down regulation of genes such as TIMP-3 and TAGLN in the WalBC cell line, which have been previously shown to be associated with highly invasive breast cancer cell lines or tumors with poor prognosis [50,51], suggests the WalBC cell line appears to exhibit a gene expression profile in common with both poorly invasive and highly invasive cell types. WalBC also exhibited overexpression of AQP3 which is associated with inflammatory breast cancer [52]. Expression of these gene in the non-invasive WalBC cell line may indicate that although these may be markers for invasive potential expression of these genes alone cannot invoke the invasive process.

It is clear the specific targets responsible for tumor progression need to be identified. A number of new targets such as hypothetical proteins, CAP4, SALL1, ZONAB, and SUMO1 were identified. These genes encoding these proteins have been found to have human ortholgoues, and with further study, may also prove to be expressed in human cancers representing possible new molecular targets for the treatment of breast cancer. In addition, MRP8, a newly discovered member of the ATP-binding cassette transporter superfamily, previously identified by EST database mining and gene prediction programming was found to be highly expressed in human breast cancer [53] and thus was identified as a putative molecular target for the treatment of breast cancer. Expression of MRP8 was also detected in the WalBC cell line and further supports this prediction demonstrating the effectiveness the WalBC cell line in the search for new targets of breast cancer therapy and treatment. Further study of these newly identified targets within human models of breast cancer may provide fresh clues in the development and progression of breast cancer which may in turn lead to new treatments and therapies.

## Conclusion

Observation of analogous gene expression profiles between human basal-like breast cancer and wallaby breast cancer has identified a common pattern of gene expression that appears to be characteristic for this type of cancer regardless of species. Therefore, the use of comparative oncology provides a useful tool to identify new potential molecular targets relevant to other species. The comparative study of breast cancer in species such as the wallaby may provide new fundamental clues to the etiology of breast cancer which may in turn lead to new treatments and therapies. In many fields unique approaches to drug discovery and design are being sort in order to unearth naturally occurring factors that may be used as potential drug therapies. One example is the use of disintegrins derived from snake venom as a potential therapeutic for treatment of breast cancer progression [54-56]. The discovery of new genes identified in wallaby breast cancer with human homologous by comparative biology may provide useful informative for further study of aspects of human breast cancer research, which may, in turn, lead to new interventions and treatment regimes.

## Abbreviations

BC: Breast Cancer; 

ECM: Extracellular Matrix; 

FCS: Fetal Calf Serum.

## Competing interests

The author(s) declare that they have no competing interests.

## Authors' contributions

JS conceived of the study, carried out the immunoassays, histological and morphologic studies, interpretation of microarray results and participated in the isolation of the cell line and in vivo studies and drafted the manuscript. SM carried out the microarray analysis and participated in collection of material and development of the cell line, proliferation assay and in vivo studies. PT carried out the data statistical analysis of the data. CL carried out normalisation of microarray data. KN performed the examination and surgery, collected material and participated in the design and coordination of the study. All authors helped to draft the manuscript, read and approved the final manuscript.
